# Multiplanar Computed Tomography of Vascular Etiologies of Acute Abdomen: A Pictorial Review

**DOI:** 10.7759/cureus.2393

**Published:** 2018-03-29

**Authors:** Muhammad Awais, Abdul Rehman, Noor U Baloch

**Affiliations:** 1 Department of Radiology, Dow University of Health Sciences (DUHS), Karachi, Pakistan; 2 Department of Medicine, Hamad Medical Corporation; 3 Department of Medicine, Rutgers New Jersey Medical School

**Keywords:** acute abdomen, emergency radiology, computed tomography, aortic dissection, aortoenteric fistula, inferior vena cava thrombosis, endoleak

## Abstract

Acute abdomen is a common presentation in the emergency department and radiologic imaging plays a pivotal role in the evaluation of such patients. Multi-detector computed tomography (MDCT) is the most commonly utilized radiologic investigation in such patients as it can be performed fairly rapidly and has excellent accuracy for diagnosing various causes of an acute abdomen. Additionally, MDCT may also reveal clues towards an alternative diagnosis that was not even suspected on the basis of a history and physical examination. Consequently, it is indispensable for radiologists to be able to accurately and efficiently recognize imaging features of disorders that may present as an acute abdomen. While gastrointestinal, hepatobiliary and genitourinary causes account for most cases of acute abdomen, vascular etiologies may also be implicated in a small—but significant—proportion of cases. Therefore, in this pictorial review, we describe the typical MDCT imaging features of various vascular etiologies that may present as an acute abdomen.

## Introduction and background

Non-traumatic acute abdominal pain is one of the most common presentations in the emergency department (ED) and an estimated 8 million patients visit the ED annually for evaluation of this complaint [1]. Clinical evaluation in the emergency department is focused on diagnosing the cause of abdominal pain. The differential diagnosis of acute abdominal pain is broad and can be related to pathologies affecting the gastrointestinal (GI), hepatobiliary, genitourinary, vascular and endocrine systems [2]. Exclusion of surgical causes and life-threatening causes of acute abdominal pain (“acute abdomen”) is of most relevance to emergency physicians and this generally requires some form of abdominal imaging [3]. In the emergency department, the radiologic modality of choice for diagnosis of acute abdominal pain is computed tomography (CT) in most cases [4]. While other imaging modalities, such as plain radiographs, ultrasonography and magnetic resonance imaging (MRI), are also utilized, CT provides exquisite anatomical details about intra-abdominal structures in a rapid manner with short turnaround times and no operator dependence. This makes it especially useful for diagnosis of acute abdomen in emergency settings [5].

Emergency abdominal CT scans are often interpreted preliminarily by trainee radiologists, especially during holidays and after usual working hours [6]. In emergency scenarios, timely and accurate interpretation of CT scans is indispensable for efficient patient care and avoidance of serious medical errors [7]. Keeping the importance of recognizing radiologic features of abdominal emergencies, a number of review articles and pictorial essays have been published regarding these entities [8]. However, in most previous reviews, vascular causes of acute abdomen have been given little attention, perhaps due to their infrequent occurrence. Over the past few decades, the number of interventional procedures performed in the United States has increased steadily and the number of iatrogenic vascular complications can be expected to have increased as well [9]. Iatrogenic vascular causes of acute abdominal pain are important entities that may be overlooked in abdominal CT scans [10]. Therefore, in the present review, we provide a pictorial review of the most common vascular etiologies of acute abdominal pain. Emergency radiologists and trainees would find this review useful and it would be prudent to routinely look for these vascular pathologies in abdominal CT scans—especially those performed for evaluation of acute abdominal pain.

## Review

1. Mesenteric ischemia

**Figure 1:**
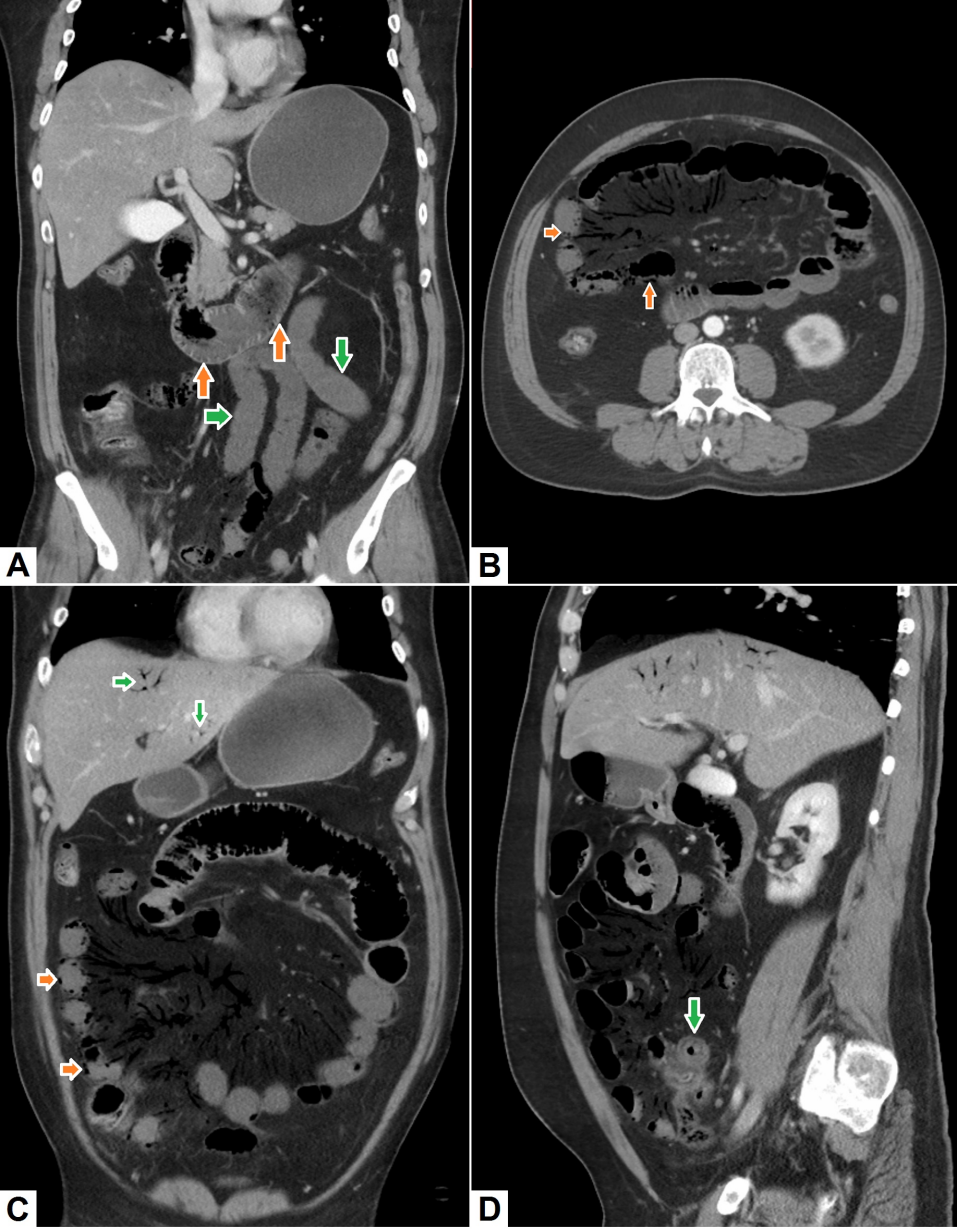
Mesenteric ischemia with pneumatosis intestinalis. (A) Coronal image shows non-enhancing jejunal loops (green arrows) as compared to the normally enhancing mucosa of the duodenum (orange arrows). (B) Axial and (C) coronal images show extensive air within the mesenteric arterial arcades. Intramural air – pneumatosis (orange arrows) – can also be seen in multiple small bowel loops. Air within the portal vein radicles (green arrows) can also be appreciated. (D) Sagittal image shows thick-walled, less enhancing small bowel loop (green arrow) with extensive air in the mesenteric vessels and within the portal radicles.

Acute mesenteric ischemia is a serious life-threatening condition. Early detection and treatment is of utmost importance as any delay in diagnosis can lead to bowel necrosis, perforation and septic shock [11]. Acute mesenteric ischemia should be suspected in elderly patients having acute abdominal pain with a prior history of left atrial thrombus, GI endovascular interventions or significant atherosclerotic disease. The typical CT features of mesenteric ischemia include mildly dilated and thick-walled bowel loops confined to a major vascular territory or its branches [12]. Mucosa of the infarcted segment is less enhancing as compared to rest of the bowel loops (see Figure 1). Other associated signs may include free air in the bowel wall (pneumatosis intestinalis), thrombus in the superior mesenteric artery or vein, and gas within the mesenteric artery, the vascular arcades or the portal venous system.

2. GI hemorrhage

**Figure 2:**
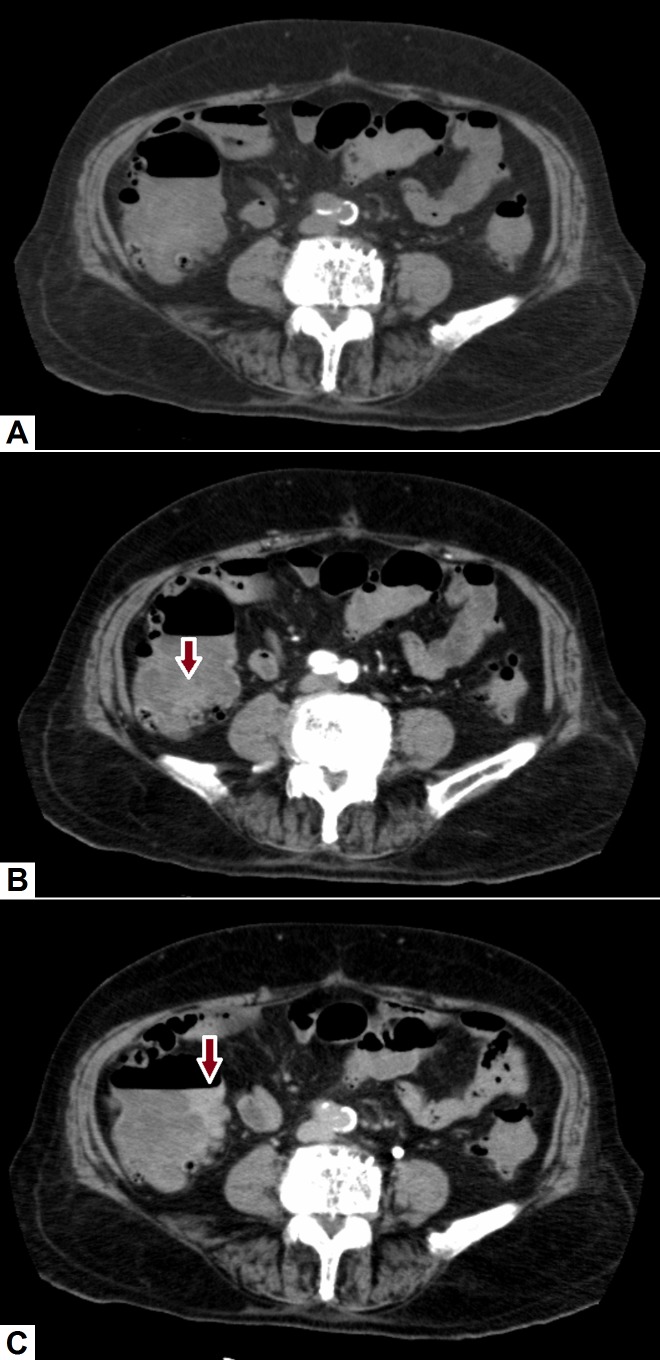
Active lower gastrointestinal hemorrhage. (A) Axial pre-contrast CT image shows slightly hyperdense material in the cecum (red arrow). (B) On arterial-phase axial image, there was an increase in the amount of hyperdense material within the cecum. (C) Axial delayed post-contrast image clearly shows active extravasation of contrast at the medial aspect of the caecum (red arrow). The patient then underwent catheter angiography for embolization of the culprit vessel (branch of ileocecal artery). CT: Computed tomography

Acute GI bleeding is another abdominal emergency with a high morbidity and mortality rate. The most common cause of acute upper GI bleed is peptic ulcer disease, while the most common cause of acute lower GI bleed, especially in the Western world, is diverticular disease [13]. In many cases of acute lower GI bleed, the underlying cause may not be found. The detection of acute GI bleed is challenging and the first line of investigation is endoscopy. However, in acute settings, it may be difficult to perform as it requires special preparation and may be unrewarding in cases of massive bleeding [14]. Red cell scintigraphy, contrast-enhanced CT and diagnostic angiography have been employed for the detection of acute GI bleed with relative advantages of each modality. However, most recent studies have suggested that contrast-enhanced CT has higher specificity as compared to red cell scintigraphy and it may also aid in planning angioembolization of the culprit vessel [15]. Many CT protocols have been employed in clinical practice including CT angiography and CT with GI bleed protocol. Most of these protocols employ an unenhanced CT examination followed by contrast-enhanced examination at fixed intervals to detect active contrast extravasation within bowel loops [16]. Figure 2 shows active contrast extravasation within the cecum detected on CT examination.

3. Abdominal aortic dissection

**Figure 3:**
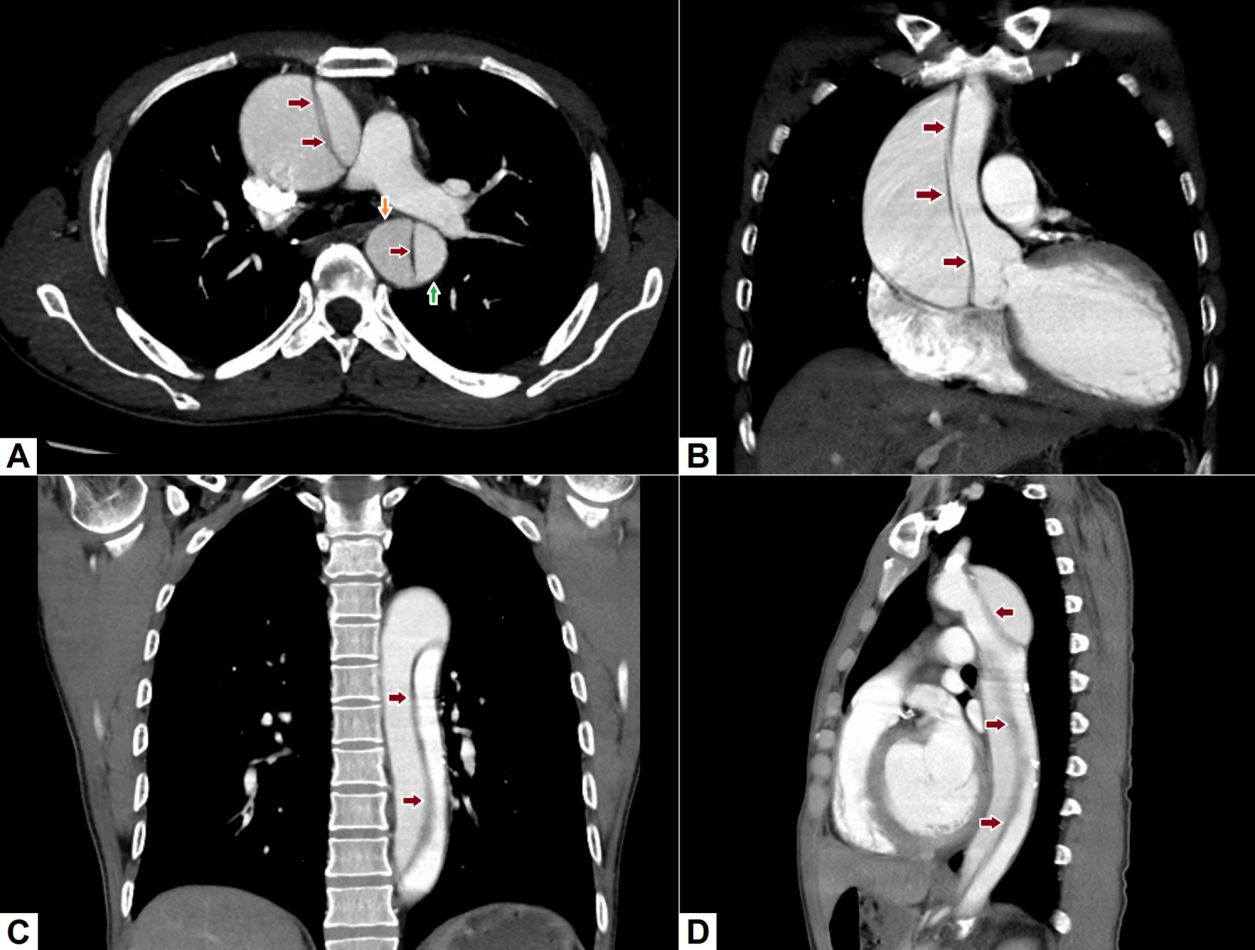
Abdominal aortic dissection. (A) Axial image demonstrates gross dilatation of the ascending aorta with an intimal flap (red arrows) identified in the lumen. The flap can also be seen in the descending aorta. There is difference in density of the contrast on both sides of the lumen. The brighter density portion represents the true lumen (green arrow), while the lower density portion represents the false lumen (orange arrow). (B), (C) Coronal images depict the extensive intimal flap involving the entire ascending and descending thoracic aorta. (D) Sagittal image demonstrating the intimal flap with true and false lumens.

Abdominal aortic dissection is another cause of acute abdomen which may have a catastrophic outcome if not diagnosed and managed promptly. Aortic dissection can be secondary to trauma, or it may be a consequence of various non-traumatic conditions, such as atherosclerotic disease, collagen vascular disease, or connective tissue disorders [17]. CT detection of aortic dissection depends on identifying an intimal flap within the aorta with the creation of a false lumen. On multiphase CT, there may be a difference in the densities of blood in the true versus the false lumen. The true lumen is typically smaller than the larger false lumen. In cases of dissection, any calcification within the wall of the aorta is usually displaced towards the center of the lumen—an important feature differentiating aortic dissection from aortic aneurysm [18]. Another important consideration in cases of aortic dissection is the extent of dissection and involvement of major branches, as it can determine the appropriate course of treatment. There may be an extension of aortic dissection into the major branches of aorta, such as the superior mesenteric or renal arteries. The false lumen can also bulge into the orifice of a major branch vessel leading to ischemia and associated complications [19]. Involvement of the ascending aorta or the aortic valvular structures necessitates immediate surgery. However, surgery is usually avoided in cases of descending thoracic or abdominal aortic dissections due to fear of inducing spinal cord ischemia [20]. Figure 3 shows a case of aortic dissection involving the entire aorta.

4. Ruptured abdominal aortic aneurysm

**Figure 4:**
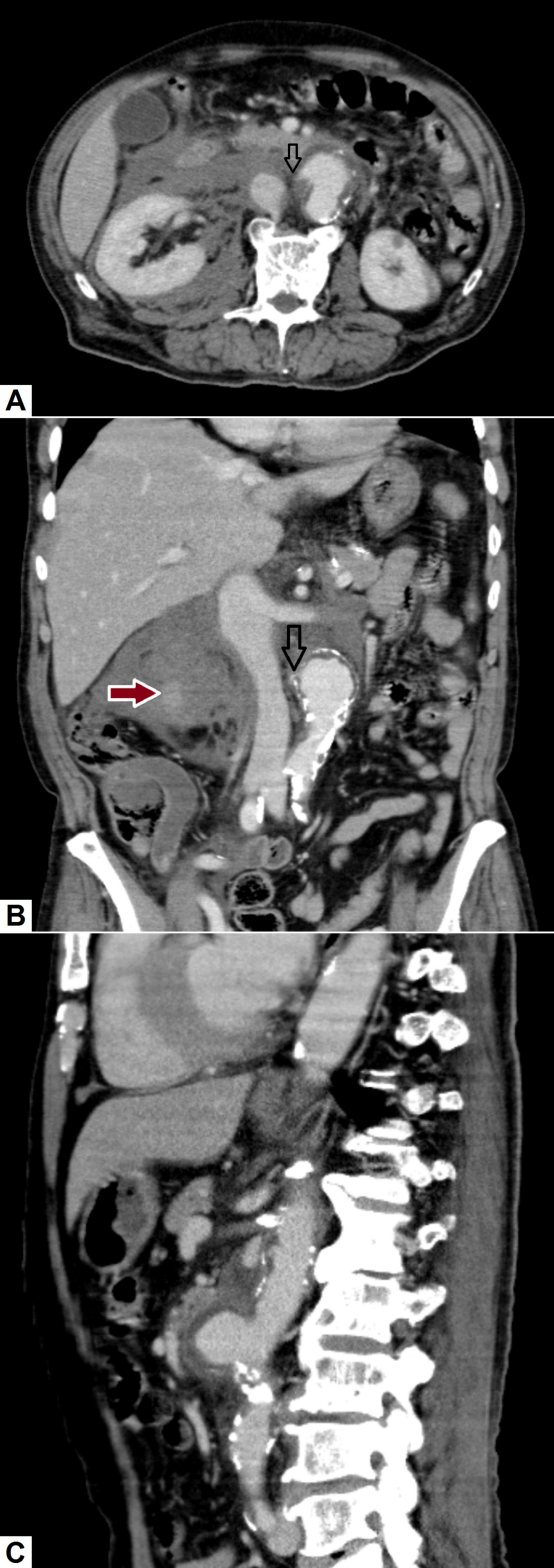
Ruptured abdominal aortic aneurysm. (A) Axial, (B) coronal and (C) sagittal images show a saccular aneurysm arising from the anterior aspect of the descending aorta with surrounding high-density fluid in the retroperitoneum (red arrow) consistent with hematoma formation. There is evidence of subtle beaking of contrast at the right margin of the aneurysm (hollow arrows) suggesting the site of leakage. The sagittal image also depicts significant atherosclerotic disease with multiple soft and calcified plaques in the abdominal aorta.

Another life-threatening disorder is rupture of an abdominal aortic aneurysm, which typically presents with severe abdominal pain radiating to the back along with possible hypotension. Aneurysms greater than 5.5 cm pose significant risk for rupture and severe abdominal pain in a patient with a known aortic aneurysm should raise suspicion for aneurysmal rupture [21]. The CT features can range from frank contrast extravasation with surrounding hematoma formation to subtle irregularity of the vascular outline or intramural hematoma [22]. Figure 4 demonstrates the CT findings in a patient with a ruptured aortic aneurysm.

5. Visceral artery aneurysms and pseudo-aneurysms

**Figure 5:**
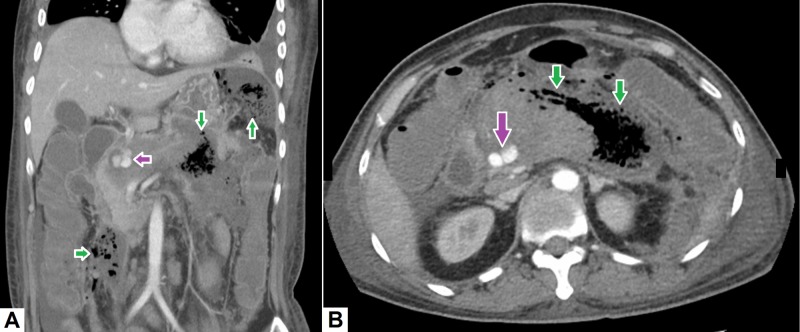
Pseudo-aneurysm of the hepatic artery. (A) Coronal image demonstrates a tri-lobed pseudo-aneurysm (pink arrow) arising near the origin of the hepatic artery proper. There is significant fat-stranding in the peri-pancreatic region with mild ascites and few large air-containing collections (green arrows) in the peritoneal cavity. (B) Axial image demonstrates pseudo-aneurysm near the head of pancreas with a large air-containing collection in the lesser sac (green arrows).

Visceral artery aneurysms are usually discovered incidentally and are often secondary to atherosclerosis and collagen vascular diseases [23]. Pseudo-aneurysms are most often a consequence of trauma, but they can also result from infective and inflammatory etiologies. Rupture of an arterial aneurysm is an emergency, which portends a worse prognosis [24]. In such cases, contrast-enhanced CT scans may reveal small saccular outpouchings from the culprit vessel with evidence of active contrast extravasation and hematoma formation. Figure 5 shows an example of a pseudo-aneurysm arising from the hepatic artery that formed as a consequence of acute necrotizing pancreatitis.

6. Tumor-related hemorrhage

**Figure 6:**
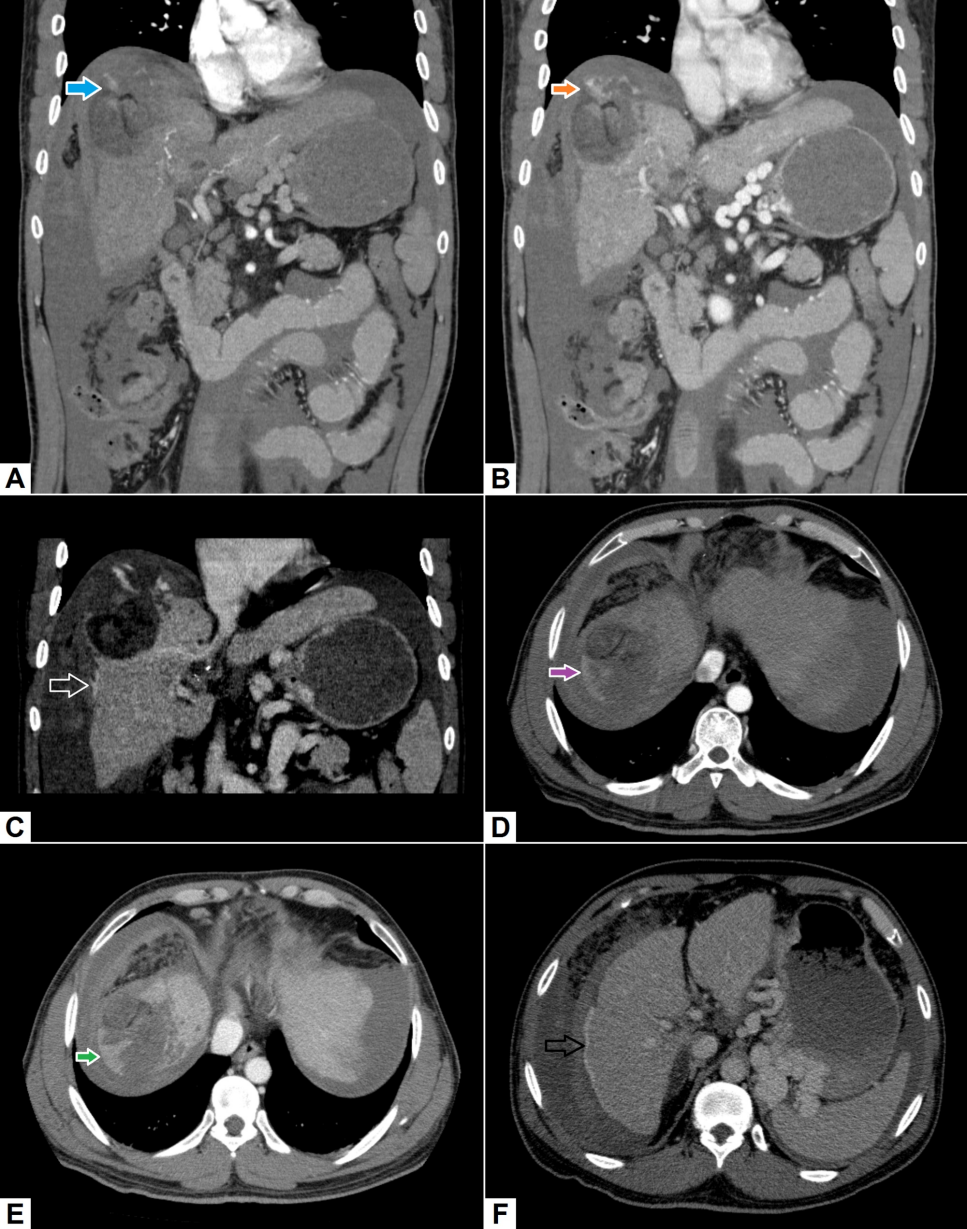
Ruptured hepatoma. (A) Coronal image shows cirrhotic liver with signs of portal hypertension evidenced by multiple large gastric varices and ascites. A large lesion in the liver can be seen in a sub-diaphragmatic location with intra-lesional vascularity in its superior aspect (arrow). (B) On delayed phase, there was active extravasation of contrast near the intra-lesional vessel (orange arrow). (C) Further delayed coronal image shows tracking of hemorrhage along the right lateral hepatic margin (hollow arrow). (D), (E) and (F) Axial images re-demonstrate a lesion in segment VIII of the liver with active extravasation of contrast within the tumor (arrows) as well as along the right margin of the liver.

Hyper-vascular tumors, such as hepatoma and angiomyolipoma, may rupture spontaneously, especially if they are exophytic [25]. Such patients present with an acute abdomen and require immediate intervention. CT is the imaging modality of choice for these patients, which will show active contrast extravasation and high-density ascites [26]. There may be a high-density focus representing a “sentinel clot” at the site of rupture. These patients should immediately undergo endovascular angio-embolization or surgery for controlling hemorrhage. Figure 6 demonstrates the CT findings in a patient who presented with an acute abdomen and was found to have a ruptured hepatoma.

7. Inferior vena cava thrombosis

**Figure 7:**
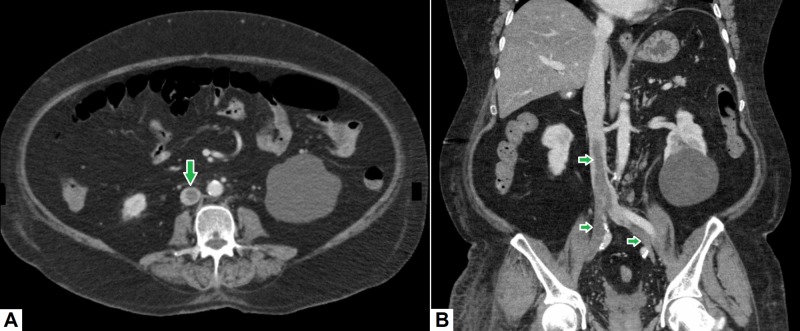
Inferior vena cava thrombosis. (A) Axial image demonstrates a rounded filling defect in the IVC representing thrombus. (B) On coronal image, the thrombus can be seen extending from the infra-renal portion of IVC into both common iliac veins. IVC: Inferior vena cava

Inferior vena cava (IVC) thrombosis is usually secondary to extension of lower extremity deep venous thrombosis, although it may occur in patients with malignancy or other hypercoagulable states [27]. In some patients with malignancies (such as renal cell carcinoma), direct extension of tumor into the IVC may result in IVC thrombosis. Moreover, in rare cases, congenital anatomic variants of the IVC may lead to thrombosis due to altered blood flow dynamics [28]. From a clinical standpoint, it is important for a radiologist to differentiate between a simple thrombus from a tumor thrombus. On CT scans, enhancement and neo-vascularity of a thrombus are suggestive of a tumor thrombus [29]. Differentiation between a bland thrombus and a tumor thrombus has implications for patient care as the presence of the latter will upstage a tumor and drastically affect patient prognosis and management. Figure 7 shows a case of a patient with IVC thrombosis extending into both common iliac veins.

The most common manifestation of IVC thrombosis is bilateral leg edema—typically, in the absence of upper limb edema—and pain. The most feared complication is pulmonary embolism, which may cause ventilation-perfusion mismatch and/or pulmonary infarction [30]. Such patients typically experience a sudden onset of dyspnea, sometimes accompanied by chest pain and/or hemoptysis. In a small proportion of cases, a saddle thrombus lodged in the main pulmonary artery may precipitate syncope or sudden cardiac arrest [31]. Consequently, in patients with IVC thrombosis, the primary team must be informed promptly in order to institute appropriate medical, endovascular and/or surgical management.

8. Renal infarction

**Figure 8:**
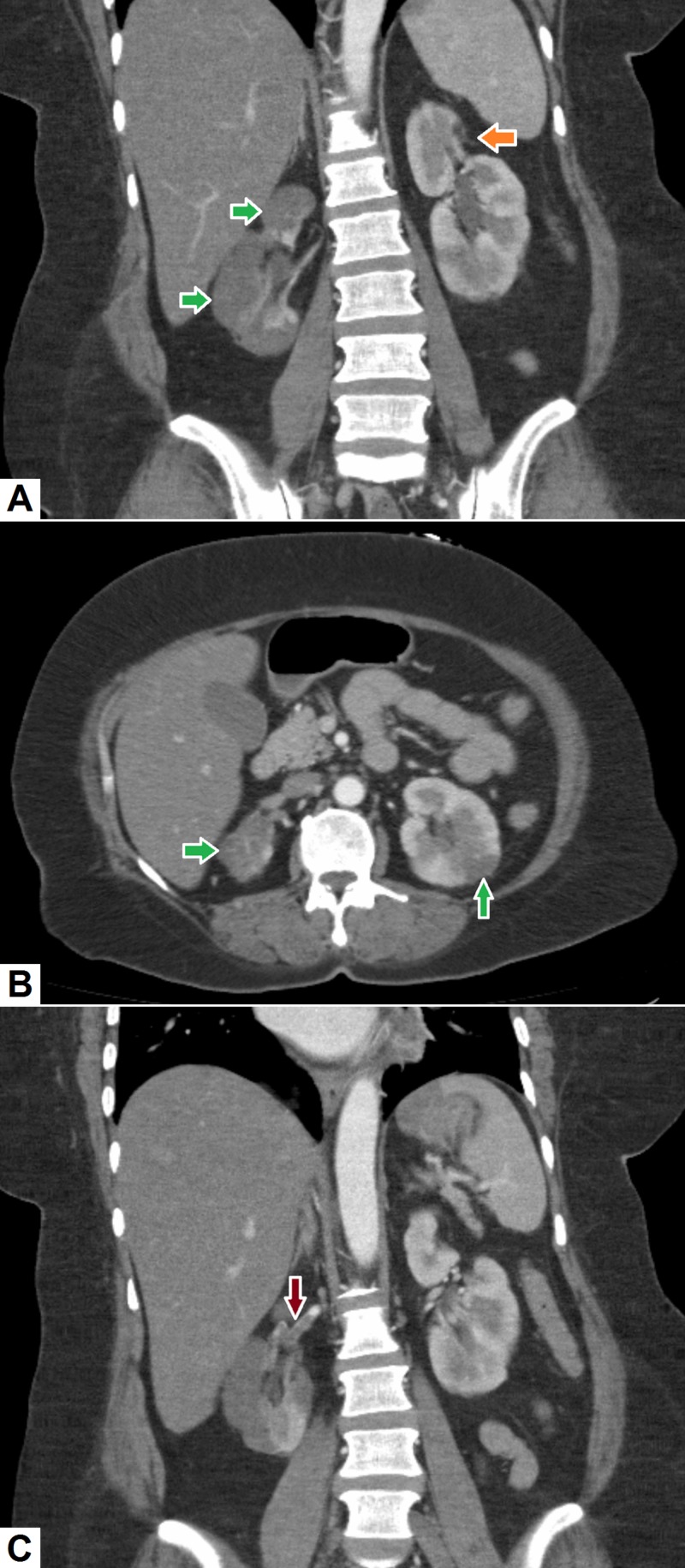
Renal infarction. (A) Coronal and (B) axial computed tomography (CT) sections demonstrate multiple low-density areas (green arrows) in the periphery of both kidneys representing infarcts. An area of focal scarring (orange arrow) can also be seen at the upper pole of left kidney on the coronal section. (C) Coronal image demonstrates a filling defect (red arrow) in the right renal artery representing a thrombus.

Renal infarcts and thrombi can be secondary to numerous diseases, such as atherosclerosis, neoplasm, thromboembolism and vasculitis. Small renal infarcts may be asymptomatic, but, large infarcts usually present with acute flank pain and hematuria [32]. CT is the imaging modality of choice for detection of renal infarction. Renal infarcts appear as hypodense, non-enhancing, wedge-shaped areas within the peripheral renal parenchyma (see Figure 8). In chronic cases, there may be associated scarring and the affected kidney may be smaller. CT may also show atherosclerotic disease or beaded appearance of renal vessels suggestive of vasculitis [33]. There may be thrombosis of the renal vein, or occasionally, a thrombus within the renal artery may also be seen.

9. Iatrogenic causes

i. Aortic Stent Graft Thrombosis

**Figure 9:**
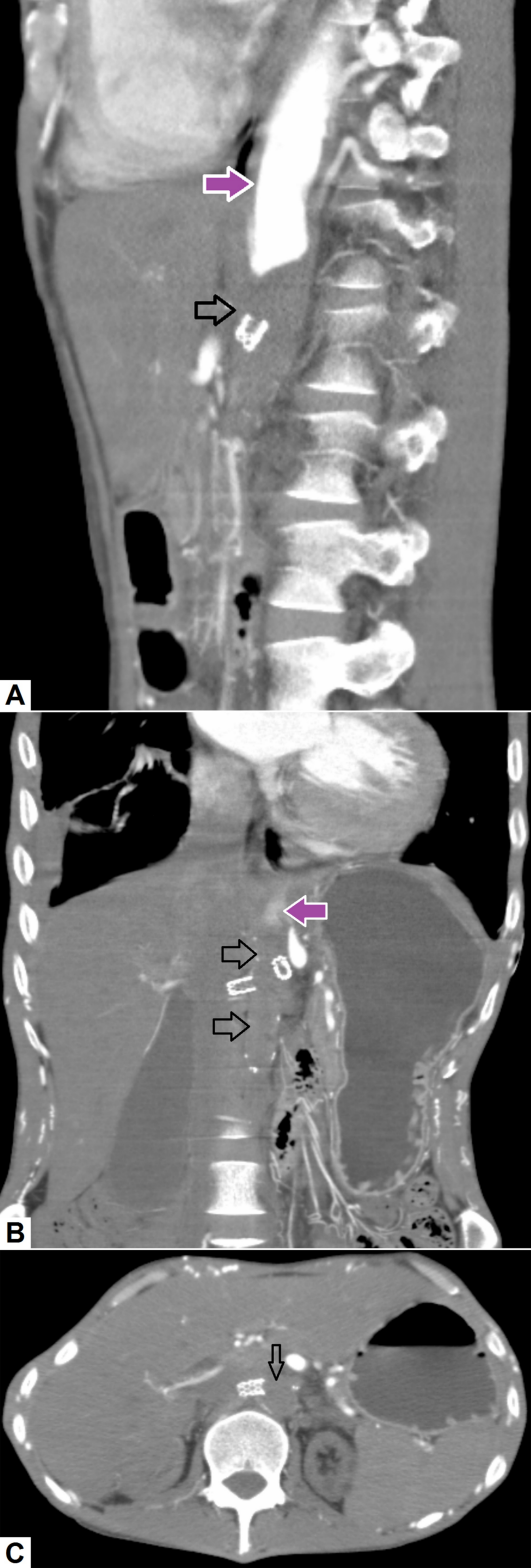
Aortic stent graft thrombosis. (A) Sagittal, (B) coronal and (C) axial images demonstrate abrupt thrombosis (hollow arrow) of the abdominal aorta just above the site of stent graft. Normal contrast can be seen in the proximal part of the aorta (pink arrow).

Aortic stent grafts are used as part of endovascular procedures to repair ruptured abdominal aortic aneurysms or dissections and have less morbidity as compared to open surgical procedures [34]. Thrombosis of the stent graft is not infrequent and is usually seen due to mechanical flow obstruction secondary to the stent graft itself. Rarely, it may be seen in patients with hypercoagulable disorders or due to non-adherence to antiplatelet therapy. Contrast-enhanced CT scan can not only depict thrombosis of the stent graft, but also delineate its extent [35]. Figure 9 depicts a case of abdominal aortic stent graft thrombosis.

ii. Aorto-enteric Fistula

**Figure 10:**
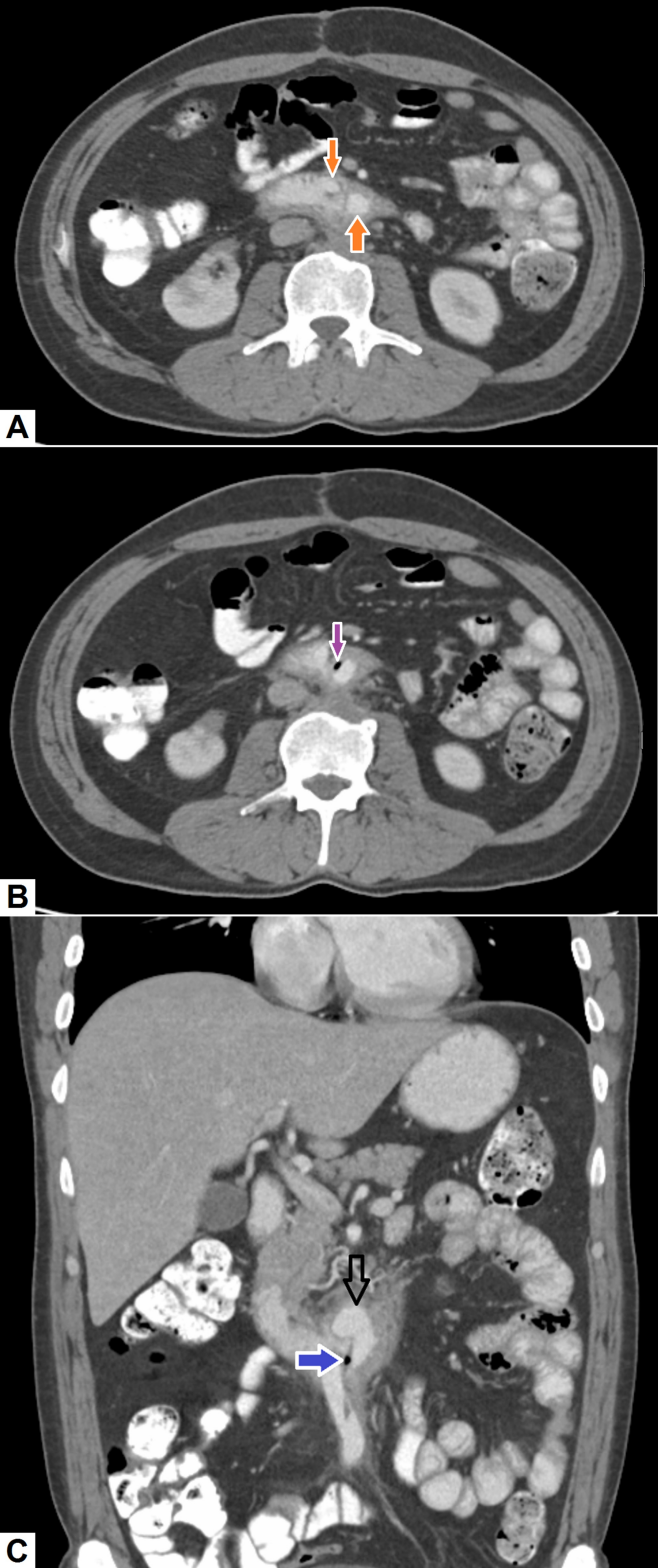
Aorto-enteric fistula. (A), (B) Axial images demonstrating aortic graft (orange arrows) within the lumen of duodenum representing aorto-duodenal fistula. A small air speck (pink arrow) is also identified in vicinity of the aortic graft. (C) Coronal image re-demonstrates the aortic graft (hollow arrow) with a tiny speck of air (blue arrow).

Aorto-enteric fistula can occur as a result of aortic reconstructive surgery with or without graft placement. However, rare instances of sporadic aorto-enteric fistula formation secondary to aortitis have been reported [36]. In cases of secondary aorto-enteric fistula, fistulous communication usually results from peri-graft infection that leads to erosion of the wall of the duodenum and eventually, the graft is seen within the duodenal lumen [37]. Patients usually present with signs of septicemia and there may be massive GI bleeding that can be fatal. CT scans can demonstrate the presence of the aortic graft within the duodenal lumen (see Figure 10). There may be associated peri-graft soft tissue, which may or may not contain specks of air [38]. Saccular mycotic pseudo-aneurysms may also be seen in the vicinity of the graft.

iii. Endoleaks

**Figure 11:**
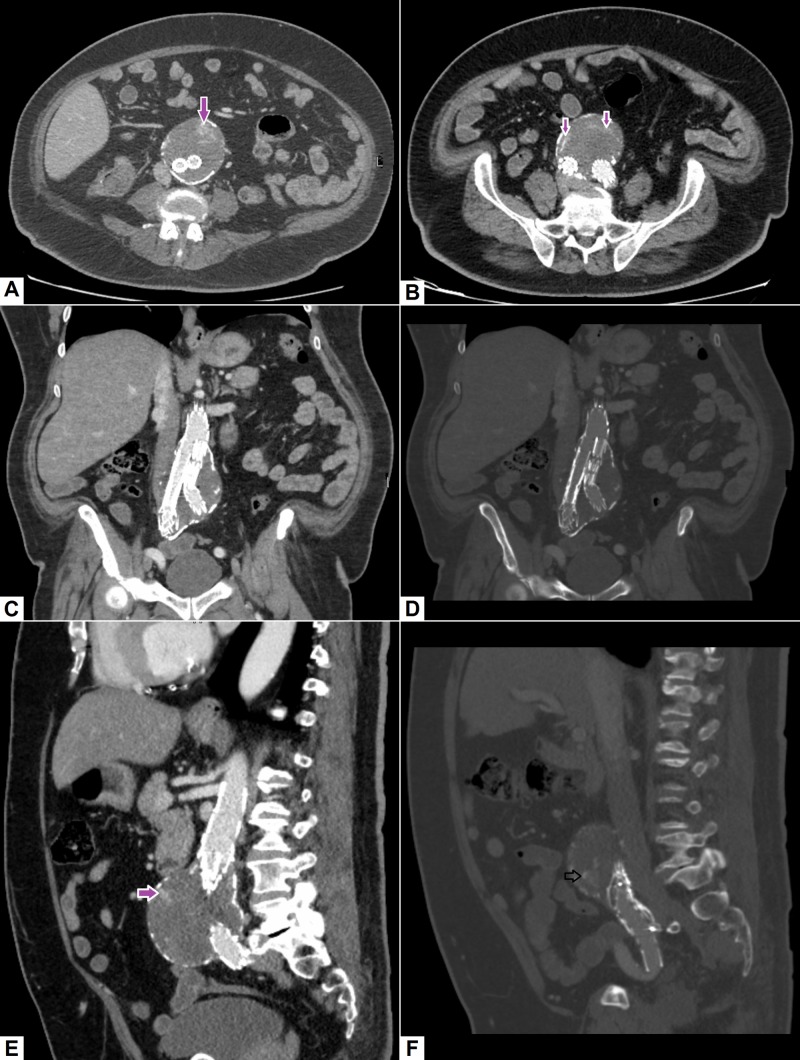
Endoleak. (A), (B) Axial and (C), (D) coronal images demonstrating aortic stent graft within the aneurysm sac. An area of contrast extravasation (pink arrow) can be identified within the aneurysm sac. (E), (F) Contrast extravasation can also be seen on sagittal images at superior and inferior aspects of the aneurysm sac representing endoleak.

Endoleak is one of the most common complications occurring after endovascular aneurysm repair [39]. Different types of endoleaks have been described. These may occur as a consequence of poor patient or stent selection, peri-aneurysmal side branches supplying the aneurysm, or secondary to the stent-graft material itself [40]. Endoleaks are usually asymptomatic, but can cause rupture of the aneurysm if the pressure within the aneurysmal sac increases substantially. For this reason, surveillance of stent grafts is required and CT is the modality of choice. Typically, multiphase CT including initial pre-contrast phase is performed, which shows extravasation of contrast into the aneurysmal sac [39, 40]. An example of an endoleak is demonstrated in Figure 11.

iv. Hemorrhage from Misplaced Stents and Catheters

Stents and catheters are often inserted in the GI, hepatobiliary and genitourinary tracts to bypass obstructions and provide a means for drainage. However, malposition of such stents and catheters can result in a number of complications including hemorrhage.

**Figure 12:**
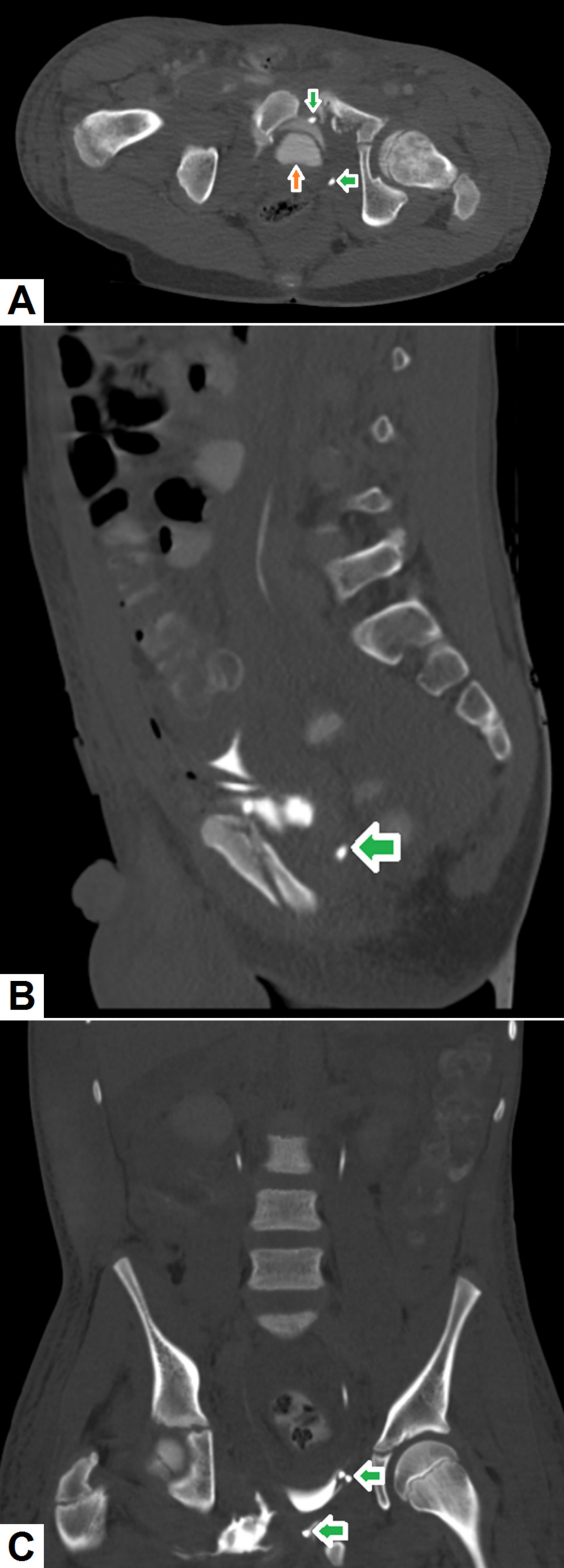
Misplaced suprapubic catheter. (A) Axial, (B) coronal and (C) sagittal images reveal a suprapubic catheter (green arrow) with its tip placed outside the urinary bladder lumen (orange arrow). Multiple fractures of the pelvic bones (secondary to a history of trauma) and surrounding hemorrhage can also be appreciated.

Misplaced drainage catheters: Drainage catheters are often placed within the peritoneal cavity to drain ascites or collections. As such catheters are usually placed under radiologic guidance, injury to neighboring structures and resultant complications are infrequent. However, in cases where a collection closely abuts a bowel loop, the drainage catheter may be inadvertently placed into a bowel loop [41]. This complication is more likely if the collection contains specks of air. While these complications may be asymptomatic in some cases, life-threatening peritonitis can ensue if bowel contents leak into the peritoneal cavity [42]. Figure 12 demonstrates the CT findings in a patient with a malpositioned suprapubic catheter.

**Figure 13:**
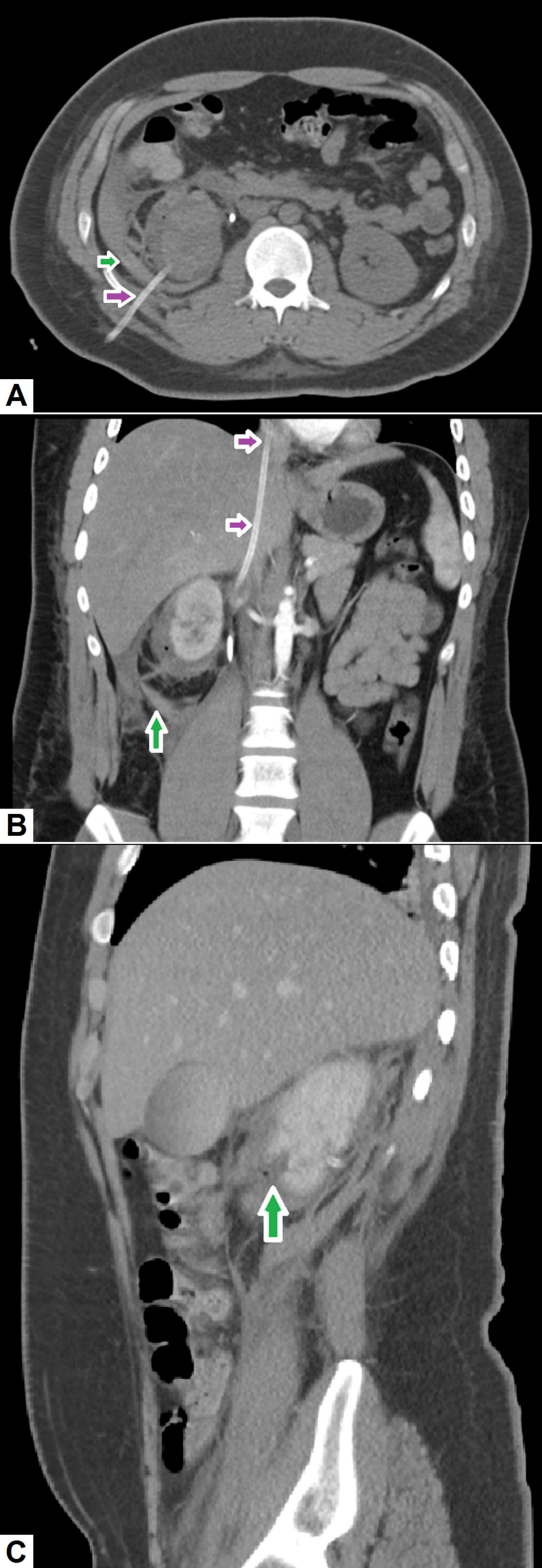
Malpositioned percutaneous nephrostomy. (A) Axial image shows a right-sided nephrostomy tube (pink arrow) with high density fluid representing hematoma around the kidney. (B) Coronal image showing a misplaced nephrostomy tube that is passing through the right renal vein and IVC into the right atrium (pink arrows). (C) Sagittal image demonstrating laceration to the lower pole of right kidney with surrounding hematoma formation. IVC: Inferior vena cava

Malpositioned percutaneous nephrostomy: Percutaneous nephrostomy is usually performed to divert the flow of urine outside the body in an obstructed renal system to avoid renal injury [43]. This procedure is useful in cases of obstructive uropathy secondary to a calculus, stricture or mass. It can also be performed in cases of ureteric injury where there is a urinary leak into the abdominal cavity. The most common complication of percutaneous nephrostomy is bleeding. Some hematuria is unavoidable and expected after a percutaneous nephrostomy procedure, but, a large peri-nephric hematoma may occur in some cases [44]. In such cases, hemorrhage usually stops by itself and the hematoma resolves with time. However, if bleeding does not stop, endovascular embolization of the renal arteries may be required. Rarely, surgery may be necessary if hemorrhage is not amenable to endovascular therapy. Figure 13 demonstrates the radiographic findings in a patient who developed severe abdominal pain and generalized weakness a few hours after undergoing a percutaneous nephrostomy procedure; abdominal CT scan revealed a large peri-nephric hematoma.

**Figure 14:**
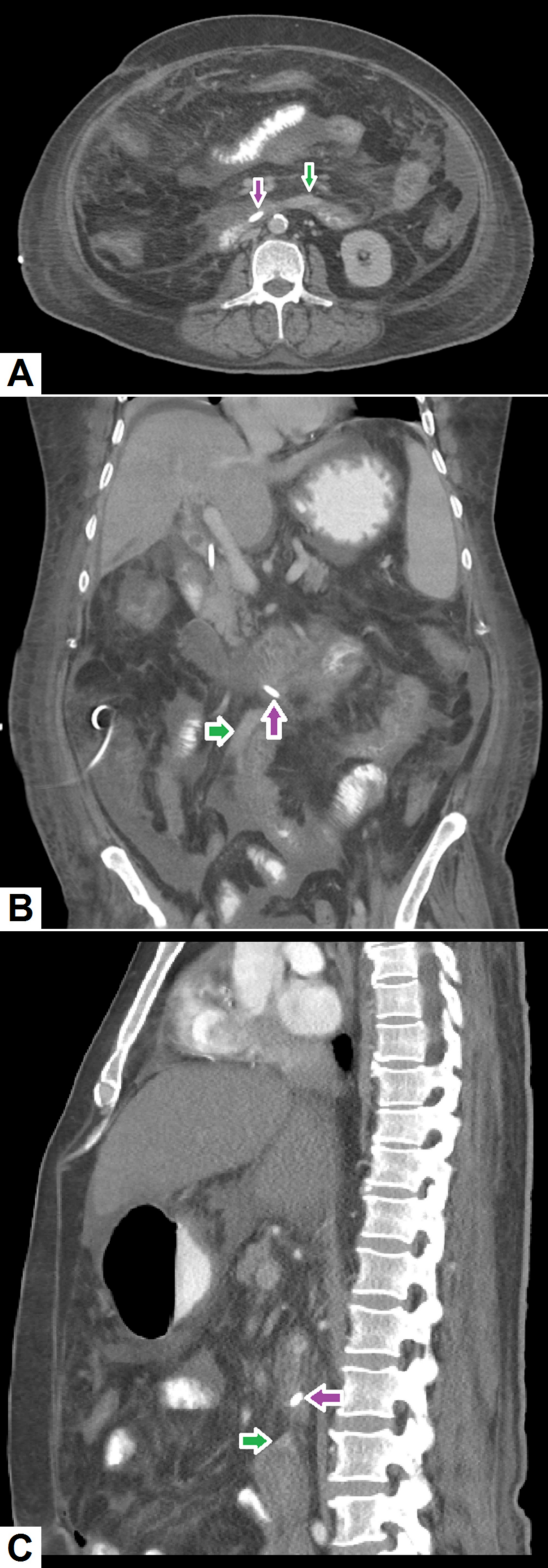
Malpositioned CBD stent. (A) Axial, (B) coronal and (C) sagittal images demonstrate the distal tip of the CBD stent (pink arrow) located outside the lumen of the duodenum with high-density fluid representing hemorrhage (green arrow) adjacent to it. CBD: Common bile duct

Malpositioned biliary stent: Placement of a common bile duct (CBD) stent is most commonly performed to bypass a biliary stricture [45]. Although infrequent, a CBD stent can inadvertently slip or malposition leading to leakage of bile into the peritoneum, which may precipitate biliary peritonitis. Bile leaks and bilomas occur more frequently as sequelae of hepatobiliary surgery, such as cholecystectomy or liver transplantation [46]. Patients often present with severe abdominal pain and sepsis. It is important to anticipate and recognize this complication early as delays in diagnosis and treatment have been associated with substantial morbidity and mortality. Although ultrasonography can be used for detection of bile leaks, its diagnostic capacity is very limited in such patients. CT scans can accurately depict peri-hepatic collections and their exact size [47]. CT may also show thickening of the CBD with a possible rent and, in cases of misplaced stents, it can accurately depict the location of the stent (see Figure 14).

## Conclusions

Acute abdomen is one of the most frequently encountered scenarios in the emergency department. Abdominal CT is the modality of choice in such cases as it can be rapidly performed and has excellent diagnostic accuracy. Vascular etiologies of acute abdomen are infrequently encountered but are often overlooked by trainee radiologists, who are usually the reviewing radiologists during after hours and on weekends. This pictorial review provided a succinct review of the most commonly encountered vascular etiologies with prototype examples of each. Recognition of vascular etiologies of acute abdomen will help radiologists and clinicians to improve patient outcomes by avoiding misdiagnosis and instituting appropriate, timely management.
